# Assessment of Patients’ Experience and Perception Toward Dentinal Hypersensitivity for Its Diagnosis and Management: A Cross-Sectional Study

**DOI:** 10.7759/cureus.35214

**Published:** 2023-02-20

**Authors:** Abdul Salam T.A., Elaf Mubarak Algharbi, Maha Ibrahim Alsane, Mayada Ibraheem Alhaji, Najed Ahmed Aldohayan, Amal Saud Albarrak, Rajkiran Chitumalla

**Affiliations:** 1 Department of Preventive Dental Science, College of Dentistry, King Saud bin Abdulaziz University for Health Sciences, King Abdullah International Medical Research Center, Ministry of National Guard Health Affairs, Riyadh, SAU; 2 Department of Preventive Dental Science, College of Dentistry, King Saud bin Abdulaziz University for Health Sciences, Riyadh, Saudi Arabia King Abdullah International Medical Research Center, Saudi Arabia Ministry of National Guard Health Affairs, Riyadh, SAU; 3 Department of Restorative and Prosthetic Dental Sciences, College of Dentistry, King Saud bin Abdulaziz University for Health Sciences, King Abdullah International Medical Research Center, Ministry of National Guard Health Affairs, Riyadh, SAU

**Keywords:** perception, prevalence, oral prophylaxis, kerala population, desensitizing agents, dentin hypersensitivity

## Abstract

Background

Dentinal hypersensitivity (DH) is a common clinical finding that varies greatly in prevalence. An appropriate impulse that detonates noxious stimuli in the pulp/dentin boundary causes the typical DH pain, which is sudden, short, sharp, and of rapid onset. The objective of this research was to ascertain the incidence of DH in Keralites and evaluate some contributory factors, along with other initiating stimuli.

Methodology

Over three months, from June 2022 to August 2022, a cluster sampling technique was employed to conduct the study in 14 districts of Kerala. Patients from five clinics from each district who reported having DH and were aged 25 to 65 years were chosen at random. Using Google Survey forms, a 20-question, closed-ended survey was mailed to patients. The data were collected, evaluated, and analyzed using SPSS Version 23.0 (IBM Corp., Armonk, NY, USA). The statistical difference in the distribution of DH and the patient’s age was determined using analysis of variance (ANOVA). A chi-square test was carried out to test the association between dentinal hypersensitivity, gender, and other parameters; *P *< 0.05 was considered to be statistically significant.

Results

Among the 2,520 persons to whom questionnaires were mailed, 2,321 responded, with an unresponsive rate of 7.89%. The study revealed a sensitivity predilection among females (54%), although statistically insignificant. Respondents aged 25 to 35 years (41.01%) were commonly reported to have DH, the age distribution of which was found to be highly significant statistically (*P *< 0.01). Teeth whitening resulted in 82.98% of the sensitivity, whereas 47.99% accepted poor oral hygiene as an additional contributory factor. DH was reported in 52.01% of the patients with gastritis and 63.03% of the patients with gingival recession. Further, blame for poor dental treatment (88.88%) and climate change (82.92%) were also found relevant. The quality of life was claimed to be afflicted in 90.99% of the patients. Desensitizing agents were used by 68.97% of the patients, with which 68.03% reported satisfaction. Of the subjects who underwent professional treatment, 87.03% reported being satisfied. Oral prophylaxis-induced sensitivity was reported in 57.99%, indicating enhanced oral health awareness among Kerala residents. There was a statistically highly significant difference in the patient distribution based on the exposure to DH, and their perception of the causes and factors affecting DH (*P *< 0.01).

Conclusions

DH is a frequently and widely prevalent dental malady. Respondents demonstrated that they were conversant with DH. The quality of life and enjoyment of daily activities are both impacted by dentin hypersensitivity, and many patients are unaware that it is a treatable illness. Various desensitizing substances are also available and can be applied quickly to sensitive areas. Patients prefer this procedure as it is simpler to apply these agents and the effects are pleasant and beneficial.

## Introduction

Dentinal hypersensitivity (DH) affects 40% of individuals and is a common oral condition [1]. An external stimulus can cause short-lived, severe mouth discomfort, which is a symptom of DH. The most frequent stimuli are hot, cold, and sweet [2]. DH, which is estimated to afflict at least one in 10 people in the general population, may also affect a person's quality of life [3,4]. According to the severity and extent of the clinical issue, current treatment approaches for DH involve either recommending an in-office (professionally applied) or over-the-counter (OTC) product. The mechanism underlying DH is hydrodynamic and based on the hydrodynamic theory, where minute fluid shifts in the dentinal tubules initiate a pain response [5]. A person's lifestyle may change due to severe hypersensitivity. Dentists must consequently have a thorough understanding of DH to correctly identify and treat the illness. Worldwide surveys of dental practitioners indicate that many are woefully unprepared to treat this illness [6].

Researchers can gather data on people's knowledge, beliefs, attitudes, and behavior, using objective methods called questionnaires. Questionnaires can be used as the foundation for cross-sectional investigations, giving descriptive information about the whole population under study. Numerous self-reported questionnaire surveys in the scientific literature gauge awareness of DH [7]. Additionally, most medical professionals admit to having some loss of confidence in their ability to effectively treat this ailment. This study is expected to increase dental professionals' understanding of this issue, which will improve patients’ oral health outcomes [8].

Treatment for DH focuses on reducing or eliminating the causes of the condition through occlusal correction, dietary recommendations, toothbrushing instructions, and desensitizing chemicals. Several in-office DH treatment approaches, including fluoride cavity varnishes, potassium-based agents, glutaraldehyde-based agents, oxalates, calcium phosphates, strontium or acetate chlorides, resin-based sealants, and laser therapy, have been described to date [9]. Studies have shown that the short-term use of sealers and restoration to lessen DH has been successful. Toothpaste is also useful, but only if used continuously for more than six months [10]. However, because they do not consider DH a serious oral health issue, the majority of the patients do not seek therapy to desensitize their teeth [11]. Continuing dental education can be a valuable supplement to dentists' limits in this area, which are a drawback [12]. It is crucial to have a management approach that involves both a preventative strategy to limit additional harm to the mouth's hard and soft tissues and a monitoring component [13]. Accordingly, this research focuses on implementing an approach that will endeavor to limit or eradicate hypersensitivity to provide the patient with a better quality of life. It also includes the relevant factors for diagnosing and controlling DH from patients’ perspectives.

## Materials and methods

A cross-sectional study was carried out to assess DH by employing a cluster sampling technique. Participants were randomly selected from five clinics from each of the 14 districts across Kerala. The study population consisted of adult patients aged 25 to 65 years, with no gender discrimination, who presented with DH in the respective clinics. Their e-mail addresses were collected from the dental clinics, and a link to the Google Survey forms was mailed to each participant along with informed consent. Patients presenting with irritation/intraoral pain/sensitivity were considered to be the inclusion criteria, whereas patients reporting a medical condition that intervenes with reliable pain (such as dementia and cognitive impairment), and chronic pain conditions like irritable bowel syndrome, temporomandibular joint (TMJ) disorders, and fibromyalgia were excluded. A 20-item questionnaire was prepared for evaluating patients' perceptions of DH. In addition, demographic information was included.

Assuming that 41% of the subjects in the population have DH, the study would have required a sample size of 2,290 for estimating the expected proportion with 5% precision, relative to the expected proportion (0.05 × 0.4 = 2% absolute precision) at a 95% confidence interval. The final sample size was estimated at 2,520, to compensate for 10% of dropouts. Therefore, a total of 2,520 questionnaires were mailed to eligible patients. Data were collected over three months, from June 2022 to August 2022. Repeated reminders were sent to the dentists monthly, to maximize the response rate. Anonymity was ensured at all levels of the study. The respondents had complete authority to reject the survey if they were unwilling. The study protocol was approved by the Institutional Ethical Committee. Google Survey forms (https://surveyheart.com/form/6342da1444872156339b710b) were used to collect data.

Statistical analysis

The results were coded, entered, and statistically analyzed using SPSS Version 23.0 (IBM Corp., Armonk, NY, USA). An independent biostatistician analyzed the data. Descriptive statistics were applied in terms of frequencies and percentages. A line diagram and pie diagrams were used to sum up the demographic variables and variables related to DH such as the patient distribution based on exposure to hypersensitivity, perception of the causes of hypersensitivity, and variable affecting DH, respectively. A pilot study was carried out by sending the survey to 20 participants to evaluate the reliability using Cronbach's coefficient alpha (value = 0.720). The statistical difference in the distribution of DH and the age of the patient was determined using analysis of variance (ANOVA). A chi-square test was carried out to test the association between DH, gender, and other parameters. *P *< 0.05 was considered to be statistically significant.

## Results

**Figure 1:**
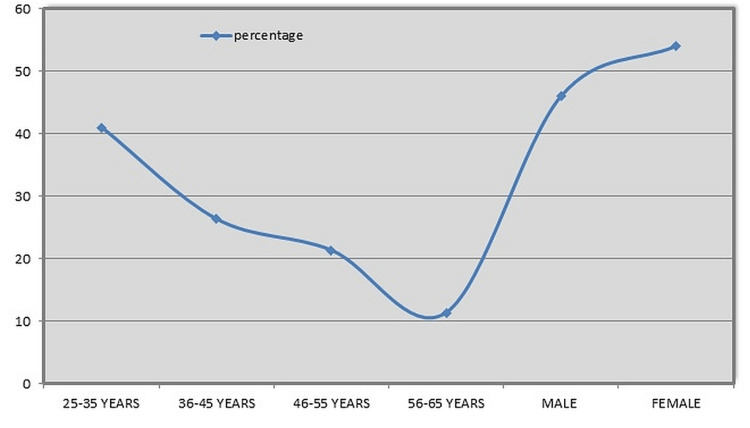
The correlation of dentinal hypersensitivity prevalence to age and sex.

The study included 1,253 males (53.98%) and 1,068 females (46.01%). Out of the total of 2,321 individuals who responded, 199 questionnaires were found incomplete, which makes the unresponsiveness rate 7.89%. The line graph depicting the age and gender distribution of the participants with DH is shown in Figure 1.

There were 952 subjects aged between 25 and 35 years, 611 between 36 and 45 years, 495 between 46 and 55 years, and 263 between 56 and 65. Thus, individuals aged between 25 and 35 years (41.01%) made up the majority of those with DH. The age distribution of DH was found to be highly significant statistically (*P *< 0.01). Analysis of the data revealed that females (54%) were more afflicted by DH than males (46%), although there was no statistical significance (*P *> 0.05). With particular foods, 77.98% of the patients had tooth discomfort. Of the total cases, 71.99% were concerned about their sensitive teeth. A sizable portion of the respondents (63.03%) reported experiencing gum issues such as a gingival recession, as the patient responds with an increase in the length of the tooth noted. Majority of the patients (52.99%) had not received any professional medical care. To relieve pain, about 68.97% of the patients used over-the-counter desensitizing medications, with which 68.03% of the patients appeared comfortable (Table 1). There was a statistically highly significant difference in the patient distribution based on exposure to DH (*P *< 0.01).

**Table 1:** Patient distribution based on their exposure to hypersensitivity.

Questions	Responses (*n *= 2,321)		
	Yes, *n* (%)	No, *n* (%)	Don't know, *n* (%)
Have you felt any type of pain in your teeth that occurs during the consumption of certain food items?	1,810 (77.98)	372 (16.02)	139 (5.98)
Are you concerned about your sensitive tooth?	1,671 (71.99)	650 (28.01)	0 (0)
Do you think the length of the tooth goes on increasing?	1,463 (63.03)	649 (27.96)	209 (9)
Have you taken any professional treatment for hypersensitivity?	1,044 (44.98)	1,230 (52.99)	47 (2.02)
Do you use any agents for dentinal hypersensitivity?	1,601 (68.97)	650 (28.01)	70 (3.01)
Are you satisfied with the agents used?	1,579 (68.03)	694 (29.90)	48 (2.06)

The cause of sensitivity, according to 71% of the patients, is decaying teeth, while 57.99% of the patients disagreed that oral prophylaxis results in hypersensitivity. Maintaining poor oral hygiene induces sensitivity, which exerted an equal influence on 47.99% of *Yes* and 48.03% of *Don't know* opinions. The influence of climate change was reported in 82.98% of the patients. While 35.97% of the patients were unaware that psychological factors may contribute to stress-related tooth abnormalities, resulting in sensitivity, and 88.88% assumed that sensitive teeth were the result of poor dental treatment. Further, 76.77% of the patients agreed that age-related sensitivity should be considered (Table 2). The patient distribution based on the perception of causes of DH was also found to be statistically highly significant (*P *< 0.01).

**Table 2:** Patient distribution according to their perception of the causes for hypersensitivity.

Questions	Responses (*n* = 2,321)		
	Yes, *n* (%)	No, *n* (%)	Don’t know, *n* (%)
Do you think a decayed tooth is a reason for hypersensitivity?	1,648 (71)	395 (17.01)	278 (11.9)
Do you believe that hypersensitivity is caused due to oral prophylaxis?	534 (23)	1,346 (57.99)	441 (19)
Do you believe that improper oral hygiene causes dentinal hypersensitivity?	1,114 (47.99)	92 (3.96)	1,115 (48.03)
Does it have any impact on exposure to climate change?	1,926 (82.98)	39 5(17.01)	0 (0)
Do you believe that any kind of stress causes tooth abnormalities that lead to hypersensitivity?	766 (33%)	720 (31.02)	835 (35.97)
Do you believe that any kind of defective dental treatment leads to hypersensitivity?	2,063 (88.88)	70 (3.01)	188 (8.09)
Do you believe that age is a factor in hypersensitivity?	1,782 (76.77)	441 (19)	98 (4.2)

Qualitative variables were viewed as a factor in sensitivity by 44.03% of the patients. DH was reported in 52.01% of the patients with gastritis. Following tooth whitening procedures, hypersensitivity was reported in 82.98% of the patients. Hypersensitivity affected patients (56.44%) taking prolonged medications. A whopping 90.99% of the patients claimed that sensitivity lowers the quality of life. Sensitivity was noted by 57.99% of the patients receiving therapy for systemic disease. Patients who received therapy from dental experts (87.03%) reported a noticeable improvement in hypersensitivity (Table 3).

**Table 3:** Patient distribution based on their perceptions of the variables affecting hypersensitivity.

Questions	Responses (*n* = 2,321)		
	Yes, *n* (%)	No, *n* (%)	Don’t know, *n* (%)
Do you think that any deleterious habits cause hypersensitivity?	1,022 (44.03)	650 (28.01)	649 (27.96)
Do you have acidity or gastritis?	1,207 (52.01)	812 (34.98)	302 (13.01)
Have you recently undergone a teeth bleaching procedure?	1,926 (82.98)	347 (14.95)	48 (2.06)
Do you have any systemic disorders?	1,346 (57.99)	813 (35.02)	162 (6.97)
Do you use any long-term medications?	1,310 (56.44)	1,011 (43.56)	0 (0)
Does the sensitivity problem affect the quality of life?	2,112 (90.99)	209 (9)	0 (0)
Do you feel any change in sensitivity after professional management of hypersensitivity?	2,020 (87.03)	186 (8.01)	115 (4.95)

The chi-square test revealed a statistically highly significant difference in the patient distribution based on their perception of the factors affecting DH (*P *< 0.01).

## Discussion

The purpose of the study was to ascertain pertinent parameters to diagnose and manage DH from patients’ perspectives. The study involved 2,321 patients from whom demographic and other relevant information were gathered and analyzed. It was evident from the findings of the study that younger people, specifically those between 25 and 35 years (41.01%), were more vulnerable to DH than those aged more than 36 years. This can be attributed to the production of secondary or tertiary dentin with advancing age. The studies of Orchardson and Collins [14], Cunha-Cruz et al. [15], and Al-Khafaji [16] provide the strongest evidence, which was congruent with the findings of this study. Age-related reductions in DH may be explained by lifetime dentin deposition, subsequent pulp atrophy, or even tooth loss in later age groups [17]. Additionally, for 82.98% of the sample population, teeth whitening treatment methods have a great impact on hypersensitivity, which was congruent with the findings of earlier studies. The study by de Paula et al. was similar to this study - a group of 40 patients given in-office bleaching operations, where 60% of the individuals admitted to having DH [18]. The incidence of such cosmetological operations is high and often affects youth.

Our study revealed that females are more impacted than males by sex preferences at rates of 54% and 46%, respectively. According to Liu et al. [19], DH affects females more frequently than males. Although the exact cause of this discrepancy is unknown, it has been hypothetically attributed to women being more conscious of oral hygiene and having greater access to general healthcare, putting them at risk of DH [20]. In our survey, 47.99% of the patients shared the view of DH is frequently associated with poor oral hygiene practices. Root exposure caused by periodontal diseases results from poor dental hygiene. Davari et al. also claimed that periodontal therapy that exposes more root surfaces may raise the risk of DH [21]. Thus, the present findings concerning 52.01% of the patients having acidity or gastritis could be explained by the generation of endogenous acid possibly resulting in DH. Mayhew et al. stated that the erosive substances containing endogenous acids reach the mouth by reflux or gastroesophageal regurgitation [22]. Patients with eating problems are more likely to be exposed to these substances. It is advised that individuals consult their doctors about any underlying systemic illnesses [23]. Yoshikawa et al. [24]. documented dental erosion in the gastroesophageal reflux syndrome (GERD) group with 24.3%. Further, 63.03% of the participants in our study reported the feeling of lengthening of their teeth, which implies gingival recession. Enamel typically covers dentine in the crown area, while periodontal tissues cover it in the root area. In these conditions, dentine is guarded against deterioration. However, periodontal tissue loss, also known as gingival recession, can expose dentine [25]. Masud et al. [26] in Malaysia, who examined the patient data with gingival recession and DH, found that both conditions had a negative physical and psychological impact on patients’ lives.

According to this study, 88.88% of the patients blame poor dental treatment for their hypersensitivity. Bubteina and Garoushi [27] further illustrated how hypersensitivity is brought on by excessive pressure used during cavity preparation, restoration leakage, incorrect bonding technique, cuspal strain, or broken restoration. Of the total patients, 68.97% were using desensitizing medications and 68.03% were comfortable with it. However, patients who received professional therapy were reported to be satisfied in 87.03% of the cases. Similar research by Blaizot et al. [28] revealed that 27% of the patients self-medicated for DH, of whom 77% were relieved. In France, 90% of those who had received therapy from a dentist were satisfied. According to our survey, 82.92% of the patients reported feeling the effects of climate change. Sensitive teeth are frequently impacted by cold climates, and occasionally in summer too. When it was sufficiently cold outside, the symptoms appeared in the autumn and winter months. Patients complained of a poorly localized, dull, and throbbing ache that began within 15 minutes after concluding an outdoor activity and returning home. According to Le Fur-Bonnabesse et al. [29], the discomfort peaked within 5-10 minutes and persisted for up to three hours, before gradually fading completely. They also observed that the use of painkillers alleviated the pain.

DH has been claimed to afflict the life quality of 90.99% of the individuals in this study. Similar findings by Barri et al. showed that 65% of the patients had significant effects on their quality of life [30]. Our findings are in line with those of Lima et al. in demonstrating the effectiveness of questionnaire-based approaches for assessing life quality in patients with DH and in identifying risk factors in determining the most appropriate course of therapy [31]. The most striking finding of this study was that the outlook of DH patients following oral prophylaxis was recorded as *no* in 57.99% of the cases. People erroneously assume that hypersensitivity is a consequence of oral prophylaxis. Here is a change that indicates enhanced oral health awareness among Keralites. One of the main limitations of the study is that results from questionnaire studies relying on the patient’s perception of the condition tend to overestimate the problem. Another problem with this study was the questionnaire method. Though previously published questionnaire studies mainly involved a face-to-face interview, this study employed a web-based survey. A further difficulty with web-based surveys is that they may be harder to validate than questionnaires administered face-to-face or with local participants. Despite these disadvantages, electronic surveys have several advantages, including the development of question scales and multiple-choice answers from qualitative exploratory interview data, the elimination of question bias through appropriate phrasing, and the incorporation of definitive and unambiguous jargon. The participants were uncomfortable providing answers in a face-to-face interview that present them unfavorably. Moreover, the study subjects may not have full knowledge of the reasons for any given answer, due to a lack of memory or even boredom. Both English language proficiency and Internet connection are prerequisites for the present research participation.

## Conclusions

This study revealed that DH is a typical oral health issue regularly seen in clinical settings. Even if the disease often has a minor-to-moderate influence on patients' quality of life, the majority of respondents stated that this impact is nonetheless unfavorable. Typically, a good first-line choice is a desensitizing home-use solution. To successfully manage the effects of DH and enhance patients’ quality of life, it is crucial to emphasize the value of ongoing professional dental care as well as pertinent information on keeping excellent oral health, such as suggestions for dietary intake and modifications to overzealous brushing habits.
